# The uniqueness of flow in probing the aggregation behavior of clinically relevant antibodies

**DOI:** 10.1002/eng2.12147

**Published:** 2020-03-15

**Authors:** Leon F. Willis, Amit Kumar, Tushar Jain, Isabelle Caffry, Yingda Xu, Sheena E. Radford, Nikil Kapur, Maximiliano Vásquez, David J. Brockwell

**Affiliations:** ^1^ School of Molecular and Cellular Biology, Faculty of Biological Sciences University of Leeds Leeds UK; ^2^ Astbury Centre for Structural Molecular Biology University of Leeds Leeds UK; ^3^ Adimab LLC Lebanon New Hampshire USA; ^4^ School of Mechanical Engineering, Faculty of Engineering University of Leeds Leeds UK; ^5^ Department of Life Sciences Imperial College London London UK; ^6^ Cornell Johnson Graduate School of Management Ithaca New York USA; ^7^ Biotheus Inc. Zhuhai Guangdong Province China

**Keywords:** aggregation, developability, extensional flow, monoclonal antibody, shear flow

## Abstract

The development of therapeutic monoclonal antibodies (mAbs) can be hindered by their tendency to aggregate throughout their lifetime, which can illicit immunogenic responses and render mAb manufacturing unfeasible. Consequently, there is a need to identify mAbs with desirable thermodynamic stability, solubility, and lack of self‐association. These behaviors are assessed using an array of in silico and in vitro assays, as no single assay can predict aggregation and developability. We have developed an extensional and shear flow device (EFD), which subjects proteins to defined hydrodynamic forces which mimic those experienced in bioprocessing. Here, we utilize the EFD to explore the aggregation propensity of 33 IgG1 mAbs, whose variable domains are derived from clinical antibodies. Using submilligram quantities of material per replicate, wide‐ranging EFD‐induced aggregation (9‐81% protein in pellet) was observed for these mAbs, highlighting the EFD as a sensitive method to assess aggregation propensity. By comparing the EFD‐induced aggregation data to those obtained previously from 12 other biophysical assays, we show that the EFD provides distinct information compared with current measures of adverse biophysical behavior. Assessing a candidate's liability to hydrodynamic force thus adds novel insight into the rational selection of developable mAbs that complements other assays.

## Graphical abstract

The development of therapeutic monoclonal antibodies (mAbs) can be hindered by their tendency to aggregate throughout their lifetime. We have developed an extensional and shear flow device (EFD), which subjects proteins to defined hydrodynamic forces which mimic those experienced in bioprocessing. Here, by comparing the flow‐induced aggregation data to those obtained previously from 12 other biophysical assays, we show that extensional flow provides distinct information compared with current measures of adverse biophysical behavior.

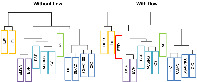

## 1 INTRODUCTION

Protein aggregation is a major problem for the biopharmaceutical industry.^1^, ^2^, ^3^ Therapeutically, the presence of aggregates may not only reduce efficacy,^4^ but can also cause adverse immune responses in vivo.^5^, ^6^, ^7^ Protein aggregation also poses significant challenges for the large‐scale manufacture of protein therapeutics,^8^, ^9^, ^10^ a sector currently dominated by monoclonal antibodies (mAbs).^11^ Aggregation can occur at any stage of a mAb's lifetime including: cell culture,^12^, ^13^ purification,^9^, ^14^, ^15^ formulation,^16^, ^17^ transportation^18^ and storage.^19^ Aggregation can thus be a major impediment to the successful translation of a candidate mAb to a commercial therapeutic. Consequently, the detection of aggregates,^20^, ^21^, ^22^, ^23^ their removal during processing^9^, ^24^, ^25^ and their general suppression,^1^, ^17^, ^26^, ^27^ has been of intense interest to academics, drug manufacturers and regulators alike. As many hundreds of sequences are generally identified during a discovery and affinity maturation campaign,^28^ so‐called “developability” assays,^29^, ^30^ are commonly deployed to aid identification of those sequences with the biophysical properties that facilitate platform manufacturing.

Two main approaches have emerged to identify developable and manufacturable sequences. First, in silico algorithms can be used to identify aggregation‐prone regions (APRs) or insoluble regions within a mAb sequence or structure, for example, Structural Aggregation Propensity,^31^, ^32^ AGGRESCAN‐3D,^33^ CamSol,^34^, ^35^, ^36^ and Solubis.^26^, ^37^ Second, a plethora of in vitro assays has been developed which probe the different biophysical properties of a mAb, such as: biolayer interferometry^38^ and self‐interaction nanoparticle spectroscopy^39^ to probe self‐association; cross‐interaction chromatography^40^ or the use of polyspecificity reagent^41^ to detect cross‐association; dynamic light scattering^42^ and standup monolayer adsorption chromatography^43^ to assess colloidal stability; as well as various spectroscopic methods to determine conformational stability.^44^, ^45^ The existence of so many methods highlights the fact that no one assay is able to identify the specific aggregation‐prone sequences that drive aggregation, especially from partially unfolded states.^46^ Furthermore, the ability of these methods to predict long‐term stability remains unclear^47^, ^48^ and, until recently, the relationship between these techniques was unknown.^49^

In an attempt to identify the design rules (akin to Lipinski's rule of five for small molecules^50^) and to understand the degeneracy (if any) between commonly used developability assays, Wittrup and colleagues used an array of 12 biophysical methods to interrogate the biophysical properties of 137 clinically relevant antibodies. These molecules were constructed by grafting the variable domain sequences of the parent molecules into a common IgG1 scaffold, enabling direct comparison of the sequences in common solution conditions.^51^ At the time of the study, 48 of these molecules were derived from approved mAbs, 42 from Phase III and 47 from Phase II. Through statistical analysis of the dataset, reinforced by direct experimental comparison of nine of the grafted IgG1s with their clinical parent molecules, they found that approved mAbs generally possessed more “desirable” biophysical properties, while those that had reached Phase‐II clinical trial (at the time of the study) generally possessed more “red flags” (biophysical behaviors deemed undesirable using a numerical threshold) than their approved counterparts.^51^ A key finding of this work was that developability assays could be divided into five distinct groups that report on: (i) nonspecific interactions using ELISA (eg, Baculovirus Particle [BVP] assay^52^), (ii) polyspecific‐ or self‐interaction (eg, affinity capture self‐interaction nanoparticle spectroscopy [AC‐SINS]^53^), (iii) loss of monomer at elevated temperature, (iv) hydrophobicity (eg, hydrophobic interaction chromatography [HIC]^54^), and (v) the expression and thermal stability of a mAb (eg, titer from transient expression in HEK293 cells).^51^

**Figure 1:**
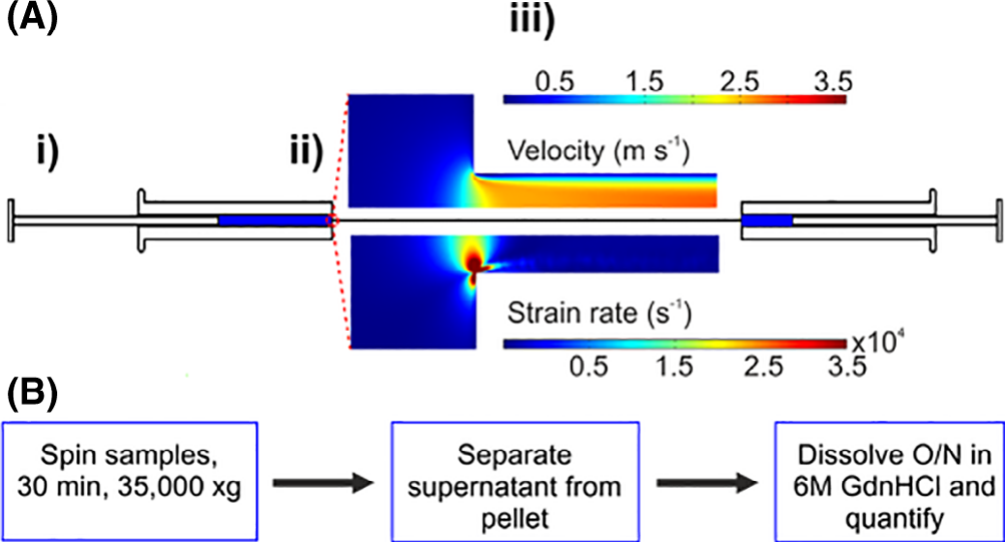
Overview of flow‐induced aggregation and its quantification. (A) Schematic of the EFD (i) 0.5 mL of protein solution is driven from one syringe to the other by a stepper motor. (ii) The two syringes are connected by a borosilicate glass capillary. The abrupt reduction in the cross‐sectional area of the flow path between the syringe and capillary (15:1) generates an extensional flow (strain rate = 11 750 s^−1^). (iii) Computational Fluid Dynamics profiles (inset) show the rapid velocity increase of the fluid from the syringe through the capillary from 8 mm s ^−1^ to 1.9 m s^−1^ (capillary wall shear rate = 52 000 s^−1^) (top), preceded by the extensional flow region (bottom). The sample then enters the second syringe chamber, prior to passage through the device in the reverse direction. (B) To quantify the effect of extensional flow, stressed, and quiescent control protein solutions are clarified by ultra‐centrifugation (30 min at 35 000*g*). Fifty microliters of the resulting supernatant and the pellet (with 50 μL supernatant above it to prevent disturbing the pellet) are dissolved overnight in buffer containing 6 M guanidine hydrochloride (GdnHCl). The fraction pelleted protein is then quantified using UV‐absorbance spectroscopy as described (Methods). *Source*: Part figure A is adapted from Reference 60 under a CC BY 4.0 license

To date, most developability assays investigate undesirable posttranslational modifications, thermal stability and specific or nonspecific interactions of the native state of a candidate mAb.^30^ It is becoming clear, however, that many aggregation pathways initiate from activated, partially unfolded metastable states that may not be detected by these techniques, especially in accelerated stability assays.^19^, ^55^, ^56^ To address this issue, we previously developed a device that exerts hydrodynamic forces, similar to those applied during bioprocessing,^9^, ^57^, ^58^ onto proteins.^1^, ^59^, ^60^, ^61^ In this method, fluid is passed between two syringes coupled via a narrow glass capillary (one fifteenth of the syringes' diameter) (Figure 1A).

The presence of a constriction generates an extensional flow field, followed by a shear flow field (Figure 1A), with these fluid fields applying hydrodynamic forces to the proteins contained within. We used this apparatus, termed the extensional flow device (EFD), to understand at a fundamental level how extensional flow induces protein aggregation through partial unfolding of proteins and demonstrated that aggregation of mAbs can be triggered by flow at concentrations as low as 0.5 mg mL^−1^.^59^ In addition, we showed that a candidate mAb with poor biophysical and pharmacokinetic properties (MEDI1912_WFL) was more sensitive to hydrodynamic flow (in terms of quantity of aggregate produced) than its derivative (MEDI1912_STT), rationally engineered to improve its biophysical characteristics.^62^ Finally, we used the EFD to identify which factors (protein concentration, sequence, buffer components, and flow conditions) are key to modulating the flow‐induced aggregation of three model IgG1 mAbs.^60^ These data show the utility of hydrodynamic force as a potential developability assay. As these studies used only three IgG1 mAbs,^59^, ^60^, ^62^ our understanding of the behavior of a broader range of therapeutic mAbs, or how the EFD relates to other methods of assessing aggregation, remained unknown.

To address these outstanding questions, we describe herein the hydrodynamic flow‐induced aggregation of 33 mAbs selected from the Jain et al dataset of 137 isotype‐matched IgG1s under common solution conditions.^51^ We show that the selected mAbs exhibit varied aggregation behavior in response to hydrodynamic stress and that this behavior cannot be predicted using currently available in silico methods. Finally, through statistical comparison of the EFD data with those of the other assays for this dataset, we show that the EFD reveals distinct biophysical information about mAbs that cannot be obtained from other assays. The EFD thus provides the user with unique information on the sensitivity of a mAb to hydrodynamic stress, which could aid the development, formulation or selection of mAb sequences most likely to succeed in a development pipeline.

## 2 MATERIALS AND METHODS

### 2.1 Protein sample selection and preparation

The 137 mAbs of the complete Jain et al dataset had previously been clustered into groups of mAbs that exhibited similar biophysical behaviors in the assays utilized.^51^ In selecting the subset of 33 proteins to be used here, it was ensured that the minimized dataset contained representatives from each cluster, weighted to reflect their respective sizes (15 from Cluster 1, six from Cluster 2 and four from each of Clusters 3, 4, and 5). The resulting set thus represents the overall spread of protein biophysical behavior observed previously (Supplementary Table I and Figure S1).^51^ The 33 Adimab protein samples, referred to as (Clinical)_33_ herein, were prepared as described previously^51^ and stored at ‐80 °C before use.

Prior to extensional flow experiments, proteins were thawed on ice and buffer‐exchanged into filtered (0.22 μm pore size, Millipore, Ireland) and de‐gassed buffer (25 mM HEPES {Sigma‐Aldrich, St. Louis}, 150 mM sodium chloride {Fisher Scientific, UK}, pH 7.3), using 3.5 kDa MW cut‐off dialysis tubes (Generon, UK). Following dialysis, samples were filtered through 0.22 μm pore size syringe filters (Biofil, China) and their concentrations determined using UV‐visible spectroscopy, using a typical 150 kDa IgG molar extension coefficient of *ϵ*_280_ ∼ 210 000 M^−1^ cm^−1^ 63 and their respective relative molecular masses (Supplementary Table II).

These stock solutions were diluted to a final concentration of 0.5 mg mL^−1^ and incubated on ice for a maximum of three hours, until required.

### 2.2 EFD experiments

The design, validation, and experimental details of the EFD are described elsewhere. 59, 60 Briefly, the device consists of two, 1 mL syringes (Hamilton, 1001 RN model) connected by a 75 mm long, borosilicate glass capillary (Sutter Instruments, B100‐30‐7.5 HP). The 15:1 difference in diameter between the syringe and capillary (inner diameters = 4.61 and 0.3 mm, respectively) generates an extensional flow at the contraction point, followed by shear flow along the capillary (Figure 1A). The syringes were washed sequentially with 2% (v/v) Hellmanex‐III solution (Hellma Analytics, Germany), 18 MΩ H_2_O and filtered buffer prior to introduction of each sample. 0.5 mL of protein solution was drawn slowly through the capillary into one syringe. Ensuring removal of air bubbles, the syringes were clamped and connected. Using a stepper motor driver slideway, the syringes were driven at a velocity of 8 mm s^−1^ for 200 passes (where one pass is defined as the emptying of one syringe and the refilling of the other) at ambient temperature. A control quiescent sample was incubated at ambient temperature for the duration of the flow experiment (200 passes at 8 mm s^−1^ takes ∼20 minutes). Following stress, the syringes were disassembled and the protein solution decanted slowly into an Eppendorf tube through the capillary. All experiments were performed in duplicate.

### 2.3 Pelleting assay to quantify insoluble protein

A pelleting assay (Figure 1B) was performed as described previously.^59^, ^60^ Briefly, 2 × 200 μL of protein sample (stressed or quiescent) were clarified by ultra‐centrifugation using a Beckmann Coulter Optima Ultracentrifuge, equipped with a TLA100 rotor (30 000 rpm [∼35 000*g*] for 30 min at 4 °C). Hundred and fifty microliters of supernatant were removed from each tube, leaving 50 μL of pellet fraction. Two hundred microliters of denaturing buffer (50 mM Tris HCl (Fisher Scientific, China), 6 M guanidine hydrochloride, pH 6 (Sigma Aldrich, Germany) was added to both the pellet fraction and to 50 μL of the supernatant. Each 250 μL (total volume) sample was then incubated overnight at 4 °C to ensure all proteins were denatured. The concentration of protein in each fraction was then determined using UV‐visible spectroscopy at 280 nm, using the extinction coefficient described above. The % protein in pellet (ie, extent of aggregation) was calculated using Equation (1):

(1) %protein in pellet=P−Sprotein0×100

Quantification of % protein in pellet where [P], [S], and [protein]_0_ are the concentration of protein in the pellet fraction, [S] supernatant fraction and initial protein solution, respectively.

The average % protein in pellet was calculated for each set of technical replicates, with the SD (error) then estimated. The final mean and error (SD) for the two biological repeats, *C* ± *δC*, was calculated according to Equation (2):

(2) δCC=δAA2+δBB2

Error propagation equation between replicates where *A* = mean of replicate *A*, *B* = mean of replicate *B*, *δA* = SD from replicate *A*, *δB* = SD from replicate *B*. The equation is rearranged and solved for *δC* (the propagated error).

### 2.4 Therapeutic aggregation profiler (TAP) analysis

TAP metric values were obtained from the TAP webserver (http://opig.stats.ox.ac.uk/webapps/newsabdab/sabpred/tap).^64^ The *V*_H_ and *V*_L_ sequences of fezakinumab and figitumumab (from Jain et al^51^) were inputted manually into the webserver. A flag was awarded to a mAb for every TAP guideline threshold that was exceeded (updated 16th June 2019, Supplementary Table III). The number of flags awarded was plotted against the % protein in pellet for the (Clinical)_33_.

### 2.5 Other in silico methods

Details regarding homology model building, isoelectric point (pI) and complementarity determining region (CDR) net charge determination, quantification of intrinsic, and structure‐corrected solubility using CamSol and APR identification using Solubis, are available in the Supplementary Methods.

*Statistical analysis of flow‐induced aggregation data*: Statistical analyses were performed on the mAb data in R (Version 3.4.4) as described previously.^51^ Red flags for each mAb were assigned based on thresholds determined previously^51^ and used to construct box plots of % protein in pellet vs the violations for each assay. Spearman's rank correlations to compare assay relatedness were performed as described previously.^51^ Briefly, the pairwise correlation matrix (excluding and including the EFD) was used as input to an agglomerative hierarchical clustering algorithm with average linkage criterion for determining cluster distance. This yielded five main assay groups: 1 (PSR {Poly‐specificity reagent}, CSI {Clone self‐interaction by biolayer interferometry}, and AC‐SINS {Affinity capture‐ self interaction nanoparticle spectroscopy}), 2 (HIC {Hydrophobic interaction chromatography}, SMAC {Standup monolayer adsorption chromatography}, SGAC‐SINS {Salt‐gradient AC‐SINS}, and CIC {Cross‐interaction chromatography}), 3 (BVP {Baculovirus particle} and multiantigen ELISA {Enzyme‐linked immunosorbent assay}), 4 (AS {Accelerated stability, using size‐exclusion chromatography to assess monomer loss}), and 5 (HEK {Expression titer in HEK293 cells} and *T*_m_ {apparent *T*_m_ by Differential Scanning Fluorimetry}). When EFD was included in the analysis, it manifested as a distinct sixth group in the clustering analysis (see main text).

To assess the robustness of the analysis to the error in the EFD measurements, bootstrapping analysis was performed. One of the two extensional flow experimental values for each of the 33 samples was picked randomly. The correlation coefficient was calculated for this set of values against the 12 developability assays and five in silico metrics. This calculation was repeated 10 000 times to obtain the median and 95% confidence intervals (Supplementary Table IV).

## 3 RESULTS

### 3.1 Subjecting mAbs to defined hydrodynamic flows reveals diverse aggregation behavior

**Figure 2:**
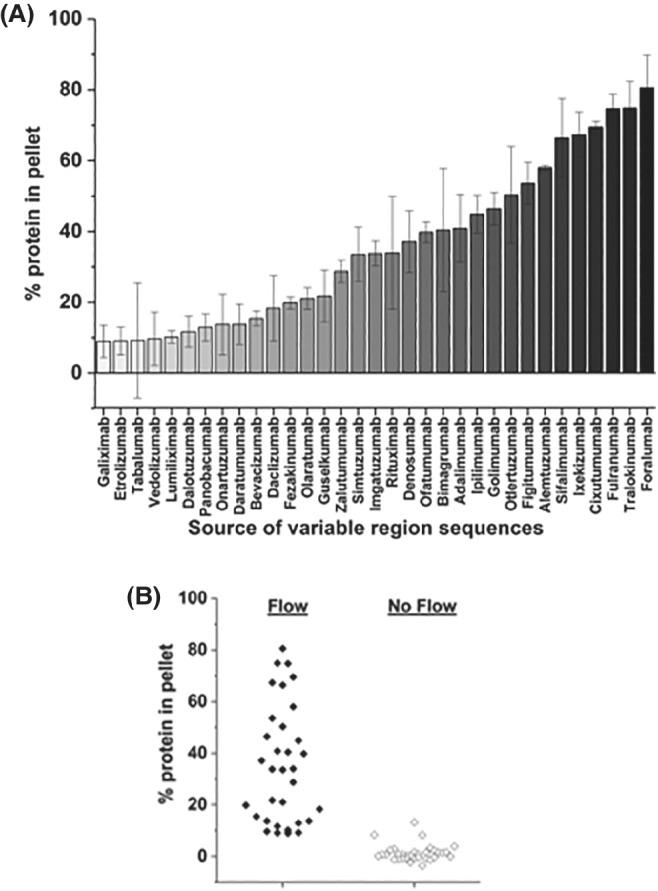
Quantification of EFD‐induced protein aggregation for the (Clinical)_33_ mAbs. (A) The percentage of mAb that was pelleted by ultra‐centrifugation after exposure to defined hydrodynamic forces. The mAb samples (at 0.5 mg mL^−1^ in 25 mM HEPES + 150 mM sodium chloride, pH 7.3) were stressed for 200 passes at a plunger velocity of 8 mm s^−1^. Samples were then analyzed using the protein pelleting assay (Methods). Error bars represent the propagated error from two independent experiments. (B) Spread of the average protein in pellet data for the (Clinical)_33_ mAbs stressed in the EFD (closed diamonds) and measured following quiescent incubation (open diamonds). The average experimental error was 7 and 3% for flow and quiescent samples, respectively

We have shown previously that mAbs are more sensitive to extensional and shear flows than proteins with other folds such as Bovine Serum Albumin (BSA), Granulocyte‐colony Stimulating Factor (G‐CSF) and β_2_‐microglobulin (β_2_m).^59^, ^60^ A subset of 33 mAbs from 137 in the original dataset^51^ were selected from across the clusters of mAbs grouped previously by their biophysical properties.^51^ Twelve of the molecules were derived from approved antibodies, nine from Phase III and twelve from phase II. This group of antibodies, dubbed the (Clinical)_33_ herein, showed varied responses in the assays employed previously (Supplementary Table I and Figure S1).^51^ The magnitude of aggregation triggered by the EFD is strongly dependent on protein concentration, strain rate (which is controlled by changing the velocity of the syringe driver) pass number (the number of times the fluid is shuttled through the capillary) and solution conditions (eg, arginine succinate buffer suppressing flow‐induced aggregation), yielding a complex response surface to flow.^60^ Here, the large number of antibodies to be studied, together with sample limitations, precluded such a detailed study. Instead, informed by previous studies,^60^ we chose a single condition (200 passes at a plunger velocity of 8 mm s^−1^ {strain rate = 11 750 s^−1^, shear rate = 52 000 s^−1^}) predicted to yield moderate aggregation levels (∼40% at 0.5 mg mL^−1^) for a “bioprocessible” mAb such as MEDI1912_STT.^60^ All 33 mAbs were stressed under common solution conditions (mAb concentration of 0.5 mg mL^−1^, formulated in HEPES‐buffered saline [Methods]) and the level of aggregation was assessed by pelleting the samples at 35 000*g* for 30 minutes, then quantifying the protein concentration in the pellet and supernatant after overnight solubilization and denaturation in 6 M guanidine hydrochloride (Figure 1B and Methods). The data (Figure 2A, B) show that the EFD‐induced aggregation of the mAbs covers a wide dynamic range (9‐81% protein in the pellet). Given the relatively large error (7% average propagated error) the extent of aggregation of each mAb was classified as low, medium or high (0‐20%, 21‐60% and >60% protein in pellet, respectively).

Few mAbs in the dataset (6/33) fall into the last, poor behavior category, a trend observed for many of the assays employed previously.^51^ This large range of flow‐induced aggregation is not observed under quiescent conditions (1 ± 3% [SD] protein in pellet, Figure 2B) and the relative levels under flow and quiescent conditions are uncorrelated (Figure S2). This demonstrates that EFD‐induced aggregation does not simply measure native‐state aggregation, which for these proteins is minimal over this relatively short timescale. Furthermore, the data suggest freeze‐thaw stresses, encountered by the molecules in the (Clinical)_33_, do not contribute to the observed aggregation behavior. Interestingly, the data show a broad response to hydrodynamic flow across the 33 samples, despite the fact the dataset comprises antibodies made with variable domain sequences sourced from mAbs that reached late stages of development or even approval. It should be noted, however, that to facilitate their expression, purification, and ease of comparison, each variable domain pair was grafted onto a common IgG1 isotype and analyzed in a single buffer. While containing sequences from “developable” molecules, these mAbs are not optimally formulated.

### 3.2 Can bioinformatics tools rationalize the EFD data?

**Figure 3:**
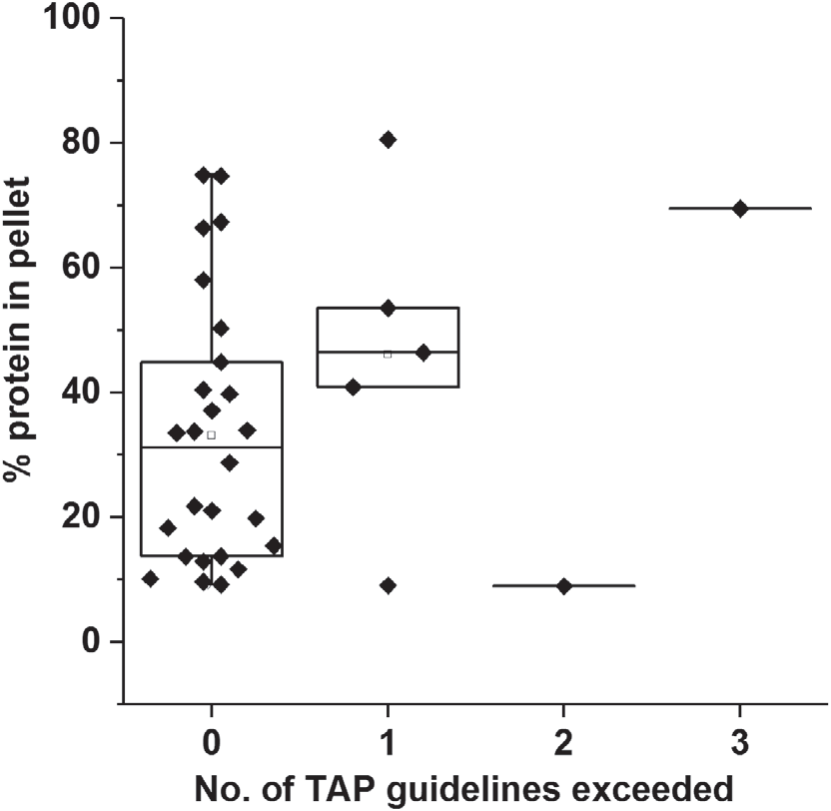
Correlation of Therapeutic Antibody Profiler (TAP) developability flags with EFD‐induced aggregation data (from Figure 2a) for the (Clinical)_33_. Box plot boundaries are the 25th and 75th percentiles, with the median bisecting the box. Only 21% of the (Clinical)_33_ dataset possessed one or more flags following TAP analysis (Methods). Some mAbs are shown to be aggregation‐prone under flow despite possessing 0 or 1 flag(s). The mAb with two amber flags is galiximab, whilst that with three amber flags is cixutumumab. Threshold values for the TAP guideline flags (updated 16th June 2019) are presented in Supplementary Table III

After showing that EFD‐induced aggregation is unrelated to quiescent behavior, we next sought to ascertain whether in silico biophysical metrics correlated with flow‐induced aggregation (Methods and Supplementary Methods). The recently developed TAP algorithm,^64^ which was partly trained on the Jain et al dataset,^51^ was used to identify undesirable CDR features in the (Clinical)_33_ which could rationalize increased aggregation under flow. No clear correlation between the number of TAP metric flags and increased aggregation in the EFD was observed (Figure 3 and Supplementary Table III).

Further in silico analyses of the (Clinical)_33_, including: pI^65^ and CDR net charge analysis,^66^ innate and structure‐corrected solubility by CamSol^34^, ^35^, ^49^ and APR identification by Solubis,^26^ were unable to discern clear correlations between the biophysical parameters of the sequences under investigation and their aggregation following stress in the EFD (Supplementary Table IV and Figures S3‐S6, respectively). We have shown previously that flow‐induced aggregation of BSA occurs concurrently with the exposure of a cysteine residue, whose side‐chain is buried in the native state.^59^ Prediction of the extent of flow‐induced aggregation by in silico approaches may thus require a better understanding of the relationship between flow fields and local protein stabilization, the study of which is in its infancy.^67^

### 3.3 How do the EFD data relate to those from alternative biophysical techniques?

We have demonstrated that EFD‐induced aggregation of the (Clinical)_33_ cannot be predicted by simple biophysical and/or structural parameters, suggesting the EFD may have a novel role in the identification of developable candidates. It has been shown recently, however, that there is redundancy amongst current developability assays.^51^, ^68^ To understand the relationship between the EFD and other methods commonly used to assess mAb developability, we compared the outputs from the EFD with the 12 biophysical techniques used previously^51^ (see Methods for full list). To allow comparison across different assays, Jain et al^51^ identified values for 10 of the methods (the bottom 10% of long‐tailed distributions, termed a red flag), below which a mAb would be identified as potentially problematic. The data for the expression titer from HEK293 cells and the apparent *T*_m_ of the Fab obtained using differential scanning fluorimetry for the 137 mAbs, were normally distributed and were thus excluded from the red flag analysis.^51^ Comparison of the distribution of EFD‐induced aggregation observed for those mAbs that passed the selection criteria for each technique, with those that failed (red‐flagged mAbs), revealed that no single assay was highly predictive of high EFD‐induced aggregation (Figure S7a‐j). Assays belonging to the poly‐specificity and ELISA clusters showed generally higher average EFD‐induced aggregation for mAbs with red flags. No strong relationship was observed between the level of flow‐induced aggregation and the number of red flags awarded to each mAb (Figure S7k), similar to the TAP data in Figure 3. Analysis of these data by Spearman's rank correlation coefficients, showed that mAbs which aggregated to the greatest extent under flow performed worst in the assays which probed poly‐specific aggregation, such as AC‐SINS, PSR, CSI and multiantigen ELISA (Supplementary Table IV), confirming the trends observed in the red flag analysis.

**Figure 4:**
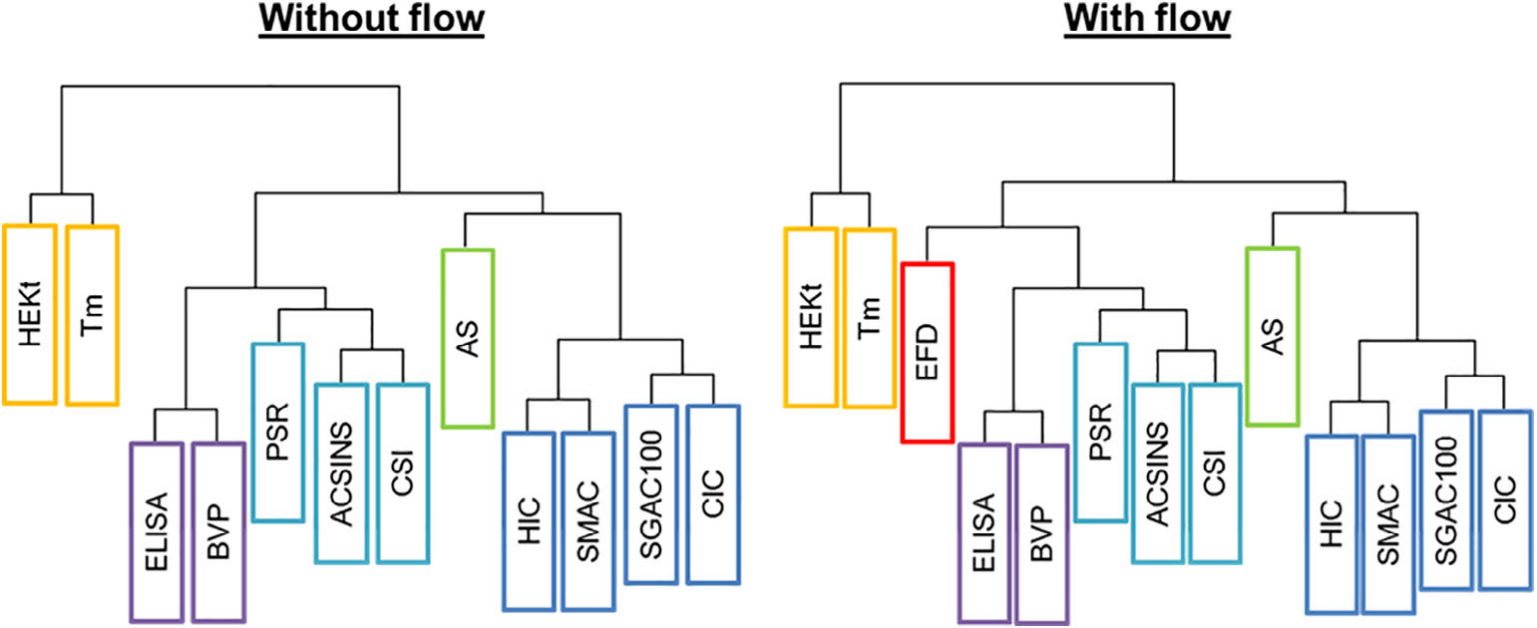
Hierarchical clustering “family trees” of assays used to probe the biophysical properties and developability of mAbs. The tree on the left shows the clustering data for the (Clinical)_33_ mAbs without the flow data. The tree on the right shows the clustering data for (Clinical)_33_ mAbs when extensional flow is included as an assay (EFD, red box). Assays are color coded according to the assay groups from Jain et al^51^ (Methods)

Finally, the relationship between the data obtained from the EFD and other developability assays was assessed using hierarchical clustering (Methods). The results (Figure 4) show that the EFD has a distinct branch on the “family tree” of assays assessed in the (Clinical)_33_ dataset. This suggests that the EFD probes a unique feature of mAbs and that protein aggregation induced by the EFD is distinct to the aggregation/association mechanisms probed by other assays.

## 4 DISCUSSION

Despite the therapeutic and commercial success of mAbs,^11^ a significant effort is still required by industrial and academic laboratories to rapidly assess and better predict the “developability” of these molecules^29^, ^49^, ^51^, ^69^, ^70^ and their more complex derivatives, such as antibody‐drug conjugates^71^ and bispecifics.^72^ Commercially, this approach should reduce cost of goods and decrease time to market. The increasing number of in vitro and in silico “developability” tools, in conjunction with the potentially large panel of molecules to screen, has led to the need to understand how different assays relate to one another, which biophysical features of molecules these assays probe and how combining these techniques increases knowledge about a mAb's developability.^3^, ^30^, ^47^, ^73^ This is particularly harmonious with the “Quality by Design” framework.^74^ Jain et al addressed this need by subjecting a panel of mAbs to a broad range of typically deployed assays to create a reference dataset.^51^ These data enable the assessment of emerging techniques and their relationship to established methods, using mAbs that vary only in the variable domains in common solution conditions (hence without formulation).^49^, ^64^, ^66^, ^70^, ^75^, ^76^

The potential for hydrodynamic forces to induce mAb aggregation has long been of concern,^9^ with fresh attention being paid recently.^77^, ^78^, ^79^, ^80^ Our efforts to address this inspired design of the EFD and its application to three IgG1s.^59^, ^60^ With the above in mind, we set out to analyze the extent of EFD‐induced aggregation for a subset of 33 mAbs from the Jain et al dataset. The (Clinical)_33_ mAbs showed wide‐ranging extents of aggregation under identical experimental conditions in the EFD. Importantly, such data were obtained using just 1 mg of material per antibody. Whilst many mAbs are administered at concentrations >100 mg/mL,^81^ we performed our measurements at 0.5 mg mL^−1^ in order to best compare the results from this study with both previous EFD studies and the Jain et al dataset. Furthermore, the use of small quantities of material in the EFD allows it to be used efficiently alongside an array of developability methods. We also emphasize that the aim of this study was to make comparisons between the EFD data and those from other datasets, as opposed to studying all the molecules in their patented formulations.

Some mAbs, with favorable “manufacturable” attributes (ie, they received 0 or 1 “red flags” in the developability assays),^51^such as those with Fv sequences from alemtuzumab and tralokinumab were found to be sensitive to hydrodynamic forces, while others (eg, Fv sequences from etrolizumab and dalotuzumab) showed the opposite behavior. We also showed that commonly employed in silico methods cannot act as a surrogate for experimental measurements of flow‐induced aggregation propensity. We have previously shown that extensional flow triggers partial unfolding of BSA,^59^ which may expose APRs that are sequestered from solvent in the thermodynamically stable native protein (Δ*G*_UN_ = 15.2 kJ mol^−1^),^82^ thereby evading detection by both CamSol and Solubis. In this regard, it is interesting to note that many of the APRs predicted by Solubis^26^ are found on the *V*_H_‐*V*_L_ interface or the CDRH3 loop (Figure S6b). Bioinformatics analysis has shown that this interface is less polar than the surface of antibody fragments.^83^ Whilst such regions are buried in static models, innate^46^, ^84^ or flow‐induced dynamics may expose APRs to the solvent, explaining the lack of correlation between the EFD and in silico predictions. The apparent lack of predictive power of these in silico methods^26^, ^34^, ^49^, ^64^ for the effects of flow may be because the mechanism of flow‐induced aggregation is different to those used to train these algorithms.

It has been suggested by others (including a reviewer) that hydrodynamic forces cannot induce aggregation.^57^, ^79^, ^85^ Whilst many agree that shear flow alone does not induce aggregation, the effects of extensional flows on proteins have largely been neglected by the field. Simon et al^86^ used a four‐roll mill device to trigger protein aggregation under extensional flows. Using an array of proteins, including three IgG1 mAbs, we have also showed that extensional (and subsequent shear) flow induced by the EFD can trigger protein aggregation and that these effects were extremely protein‐ and flow‐field specific.^59^, ^60^ More recently, EFDs of similar design to that employed in our study have been constructed and mAb aggregation observed under extensional and shear flows.^87^, ^88^ These and other studies^57^, ^79^, ^85^, ^89^, ^90^, ^91^, ^92^, ^93^ have shown that the presence and chemical composition of interfaces and presence of localized high protein concentrations within flow devices also contribute to the aggregation cascade. Others have suggested that the friction between the solid components of flow devices, for example, Couette cylinders^89^ or plungers and syringe barrels, can also induce aggregation. The relative importance of stain rate, surface chemistry, and friction for EFD‐induced protein aggregation is beyond the scope of this study.

The aim of this study was not to understand the molecular mechanism of EFD‐induced aggregation but to investigate the relationship between the EFD and other commonly employed developability assays. The results presented show that the EFD induces a wide‐range of aggregation response that is not correlated with other metrics of developability as demonstrated by hierarchical clustering. In response to a reviewer, we have used bootstrapping analysis to show that the clustering is statistically significant, despite an average error of ±7% for the EFD data. As such, the EFD may reveal a new critical quality attribute that must be met for a candidate mAb to proceed to development.

The EFD could thus allow poorly behaving candidates to be identified early in the development pipeline. Moreover, by varying the flow conditions and the solution properties, the EFD can be used to select mAbs that are resilient to flow stresses.^60^ This information could be taken into late‐stage development, allowing manufacturing conditions and/or formulations to be changed to suppress the aggregation of the molecule under hydrodynamic forces.^60^ Such information cannot be gleaned from currently available assays or in silico methods. Similarly to other assays,^19^, ^55^ the ability to predict long‐term stability and the relationship between the EFD data and accelerated stability is still to be determined. Overall, the EFD could help industry screen molecules and accelerate the selection of promising candidates to be taken forward for biomanufacture.

## CONFLICT OF INTEREST

The authors declare no conflicts of interest.

## AUTHOR CONTRIBUTIONS

Leon F. Willis contributed to the conceptualization, data curation, formal analysis, investigation, methodology, writing of the original draft, review, and editing. Amit Kumar contributed to the formal analysis, investigation, methodology. Tushar Jain contributed to the formal analysis, methodology, writing of the original draft. Isabelle Caffry contributed to the resources. Yingda Xu contributed to the resources. Sheena E. Radford contributed to the project administration, supervision and editing. Nikil Kapur contributed to the methodology, project administration, supervision and editing. Maximiliano Vásquez contributed to the project administration, supervision and editing. David J. Brockwell contributed to the conceptualization, project administration, supervision and editing.

## Supporting information

Data S1 Supporting information

## Endnote

1 Reference 61 is the PhD thesis of LFW, where some of this work is presented. See reference list for link.
