# Supplementary material for: The uniqueness of flow in probing the aggregation behavior of clinically relevant antibodies
Source: Eng Rep. 2020 Mar 15;2(5):e12147. doi: 10.1002/eng2.12147 (PMC8638667; doi:10.1002/eng2.12147)
Supplement: Supplementary file 1 — Data S1 Supporting information [file ENG2-2-e12147-s001.docx]

**Supplementary Information for**

**The uniqueness of flow in probing the aggregation behaviour of clinically relevant antibodies**

Leon F. Willis ^1,2^, Amit Kumar ^1,2^, Tushar Jain ^3^, Isabelle Caffry ^3^, Yingda Xu ^3^, Sheena E. Radford ^1,2^, Nikil Kapur ^4^, Maximiliano Vásquez ^3^ and David J. Brockwell* ^1,2^

**Supplementary Methods**

***Hierarchical clustering:*** Full details regarding the clustering of the antibodies in the original dataset are given in Jain et al., 2017.^1^ The clusters, shown in Figure S1, represent groups of antibodies with similar biophysical properties. These clusters were used to inform the selection of the (Clinical)_33_ used here.

***Homology model building****:* The amino-acid sequences of the variable domain pairs (V_H_ ­+ V_L_) for all (Clinical)_33_ proteins were input as FASTA sequences into the ABodyBuilder^2^ webserver (<http://opig.stats.ox.ac.uk/webapps/sabdab-sabpred/Modelling.php>). The output models were saved as .pdb files for editing in PyMol or UCSF Chimera (see below).

***pI, CDR net charge and intrinsic solubility calculations:*** The pI and CDR net charges (CDRs defined using Kabat numbering from the ABodyBuilder models) were calculated using the ProtParam^3^ webserver (<https://web.expasy.org/protparam/>), with each His residue assigned a charge of +0.1.^4^ The intrinsic solubility at pH 7 of the variable domains (V_H_ + V_L_) for each mAb (note: the scaffold region (constant domains) is identical for all of the mAbs in this study) was calculated using the CamSol^5^ webserver (<http://www-mvsoftware.ch.cam.ac.uk/>).

***Structure-corrected solubility score from CamSol:*** The homology models for the (Clinical)_33_ were input into the CamSol^5^ structure-corrected webserver (<http://www-mvsoftware.ch.cam.ac.uk/>). The .pdb files were run through the ‘PDB cleaner’ script on the webserver to facilitate their use in the CamSol calculation. The solubility scores were then computed using the default settings of pH 7 and a patch radius of 10 Å. Output structures, highlighting regions of insolubility, were visualized using UCSF Chimera (University of California, San Francisco).^6^

***APR identification in Solubis:*** To identify APRs in each variable domain pair within the (Clinical)_33_, the homology models constructed in ABodyBuilder,^2^ were input into the Solubis^7,8^ webserver (<http://solubis.switchlab.org/>). To identify solvent-exposed APRs, Solubis locates such residue stretches using the TANGO algorithm^9^ (higher values denote an increased propensity to aggregate), and assesses their importance to native-state aggregation by evaluating the thermodynamic stability of this region (*ΔG^contrib^)* using FoldX^10^ (where a *ΔG^contrib^* ≤ 0 is favorable). These are used to compute weighted ‘Solubis’ scores,^7^ by normalizing the *ΔG^contrib^* scores for all APRs (*ΔG^contrib^* -5 to +5 kcal/mol) from 0 to 1, then multiplying these values by the β-aggregation propensity score from TANGO.

***Statistical analysis of in silico data:*** Spearman’s rank correlation coefficients were obtained for the *in silico* data and the % protein in pellet using OriginPro 2016.

**Supplementary Table I: Hierarchical clustering of (Clinical)_33_ proteins from Jain et al.** ^1^

| **Source of variable region**  **sequences** | **Cluster number** | **Group 1 Flag** | **Group 2 Flag** | **Group 3 Flag** | **Group 4 Flag** | **Total number of flags** |
| --- | --- | --- | --- | --- | --- | --- |
| adalimumab | 1 | 0 | 0 | 0 | 0 | 0 |
| alemtuzumab | 1 | 0 | 0 | 0 | 0 | 0 |
| daclizumab | 1 | 0 | 0 | 0 | 0 | 0 |
| daratumumab | 1 | 0 | 0 | 0 | 0 | 0 |
| lumiliximab | 1 | 0 | 0 | 0 | 0 | 0 |
| ofatumumab | 1 | 0 | 0 | 0 | 0 | 0 |
| onartuzumab | 1 | 0 | 0 | 0 | 0 | 0 |
| otlertuzumab | 1 | 0 | 0 | 0 | 0 | 0 |
| panobacumab | 1 | 0 | 0 | 0 | 0 | 0 |
| rituximab | 1 | 1 | 0 | 0 | 0 | 1 |
| sifalimumab | 1 | 0 | 0 | 1 | 0 | 1 |
| tabalumab | 1 | 1 | 0 | 1 | 0 | 2 |
| tralokinumab | 1 | 1 | 0 | 0 | 0 | 1 |
| vedolizumab | 1 | 0 | 0 | 0 | 0 | 0 |
| zalutumumab | 1 | 0 | 0 | 0 | 0 | 0 |
| bimagrumab | 2 | 1 | 1 | 1 | 1 | 4 |
| denosumab | 2 | 0 | 0 | 0 | 0 | 0 |
| figitumumab | 2 | 1 | 1 | 1 | 0 | 3 |
| foralumab | 2 | 1 | 1 | 1 | 0 | 3 |
| fulranumab | 2 | 0 | 0 | 1 | 0 | 1 |
| imgatuzumab | 2 | 0 | 0 | 1 | 0 | 1 |
| ipilimumab | 3 | 1 | 1 | 0 | 0 | 2 |
| fezakinumab | 3 | 1 | 1 | 0 | 0 | 2 |
| golimumab | 3 | 1 | 1 | 0 | 0 | 2 |
| galiximab | 3 | 0 | 1 | 0 | 0 | 1 |
| bevacizumab | 4 | 0 | 1 | 0 | 1 | 2 |
| etrolizumab | 4 | 1 | 0 | 1 | 1 | 3 |
| guselkumab | 4 | 1 | 0 | 1 | 1 | 3 |
| olaratumab | 4 | 1 | 0 | 0 | 1 | 2 |
| cixutumumab | 5 | 1 | 1 | 1 | 1 | 4 |
| dalotuzumab | 5 | 1 | 0 | 1 | 1 | 3 |
| ixekizumab | 5 | 1 | 0 | 1 | 1 | 3 |
| simtuzumab | 5 | 1 | 0 | 1 | 1 | 3 |

The cluster number for each mAb is derived from the analysis of Jain et al. to yield the data shown in Figure S1. The four assay groups in Jain et al. are: 1 (PSR {Poly-specificity reagent}, CSI {Clone self-interaction by Bio-layer interferometry}, AC-SINS {Affinity capture- self interaction nanoparticle spectroscopy}), 2 (HIC {Hydrophobic interaction chromatography}, SMAC {Standup monolayer adsorption chromatography}, SGAC-SINS {Salt-gradient AC-SINS} and CIC {Cross-interaction chromatography}) 3 (BVP {Baculovirus particle} and ELISA {Enzyme-linked immunosorbent assay}) and 4 (AS {Accelerated stability, using size-exclusion chromatography to assess monomer loss}). Exceeding a ‘poor behavior’ threshold for an assay within a group results in that protein being awarded a ‘red flag’. Generally, the number of red flags assigned to each protein increases as cluster number increases. Full details of this analysis are provided in Jain et al.^1^

**Supplementary Table II: Molecular weights of the (Clinical)_33­_ proteins**

| **Source of variable region**  **sequences** | **Molecular Weight (Da)** |
| --- | --- |
| adalimumab | 148,079 |
| alemtuzumab | 148,652 |
| bevacizumab | 149,132 |
| bimagrumab | 145,354 |
| cixutumumab | 148,964 |
| daclizumab | 146,720 |
| dalotuzumab | 148,852 |
| daratumumab | 147,903 |
| denosumab | 147,797 |
| etrolizumab | 146,944 |
| fezakinumab | 147,302 |
| figitumumab | 149,084 |
| foralumab | 148,164 |
| fulranumab | 148,464 |
| galiximab | 147,805 |
| golimumab | 149,543 |
| guselkumab | 146,450 |
| imgatuzumab | 147,883 |
| ipilimumab | 147,933 |
| ixekizumab | 149,357 |
| lumiliximab | 150,312 |
| ofatumumab | 148,574 |
| olaratumab | 152,607 |
| onartuzumab | 149,813 |
| otlertuzumab | 147,295 |
| panobacumab | 148,740 |
| rituximab | 147,130 |
| sifalimumab | 146,838 |
| simtuzumab | 148,302 |
| tabalumab | 149,107 |
| tralokinumab | 147,052 |
| vedolizumab | 149,491 |
| zalutumumab | 149,121 |

Proteins are listed alphabetically. A common IgG1 molar extinction coefficient of 210,000 M^-1^ cm^-1^ at 280 nm was used for all proteins when determining protein concentrations by UV-visible spectrophotometry (Methods).

**Supplementary Table III: Therapeutic Antibody Profiler (TAP)** ^11^ **Guidelines (updated 16^th^ June 2019)**

| **Property** | **Amber Region** | **Red Region** |
| --- | --- | --- |
| **Total CDR Length (L)** | 39 ≤ L ≤ 42 | L < 39 |
|  | 55 ≤ L ≤ 60 | L > 60 |
| **Patches of Surface Hydrophobicity** | 88.2933 ≤ PSH ≤ 100.788 | PSH < 88.2933 |
|  | 158.470 ≤ PSH ≤ 179.175 | PSH > 179.175 |
| **Patches of Positive Charge (PPC)** | 1.255 ≤ PPC ≤ 3.162 | PPC > 3.162 |
| **Patches of Negative Charge (PNC)** | 1.846 ≤ PNC ≤ 3.463 | PNC > 3.463 |
| **Structural Fv Charge Symmetry Parameter (SFvCSP)** | -20.40 ≤ SFvCSP ≤ -6.194 | SFvCSP < -20.40 |

The guideline values above were set by models for 377 post Phase-I therapeutics. Full details of these molecules and the metrics are available online (<http://opig.stats.ox.ac.uk/webapps/newsabdab/sabpred/tap>), with full details of the algorithm given in Raybould et al., 2019.^11^

**Supplementary Table IV: Spearman’s rank correlation analysis of the EFD with *in silico* metrics and twelve other ‘developability’ assays** ^1^**.**

| **Metric** | **Spearman Rank Correlation with average % protein in pellet from two independent experiments** | | **Spearman Rank Correlation from bootstrapping analysis** | |
| --- | --- | --- | --- | --- |
|  | Coefficient | p-value | Median coefficient | 95% confidence intervals |
| Solubis | -0.19 | - | -0.20 | -0.26, -0.13 |
| CamSol – structure corrected | 0.18 | - | 0.16 | 0.09, 0.23 |
| CamSol – intrinsic | 0.22 | - | 0.21 | 0.12, 0.28 |
| pI | 0.32 | 0.08 | 0.31 | 0.25, 0.36 |
| CDR Net Charge, pH 7 | 0.21 | - | 0.20 | 0.14, 0.26 |
| HEK Titer | -0.05 | - | -0.06 | -0.15, 0.02 |
| T_m_ | -0.09 | - | -0.10 | -0.19, -0.02 |
| ELISA | 0.30 | 0.09 | 0.31 | 0.26, 0.38 |
| BVP ELISA | 0.14 | - | 0.16 | 0.09, 0.23 |
| PSR | 0.29 | 0.10 | 0.28 | 0.21, 0.34 |
| ACSINS | 0.52 | 0.002 | 0.51 | 0.45, 0.56 |
| CSI | 0.40 | 0.02 | 0.39 | 0.34, 0.44 |
| AS | -0.13 | - | -0.13 | -0.2, -0.06 |
| HIC | -0.05 | - | 0.05 | -0.13, 0.03 |
| SMAC | -0.08 | - | -0.06 | -0.13, 0.01 |
| SGAC-SINS | 0.21 | - | 0.20 | 0.13, 0.27 |
| CIC | 0.29 | 0.097 | 0.28 | 0.20, 0.35 |

Data derived from *in silico* analyses are shaded. All *p*-values less than 0.1 are listed in the table.

**
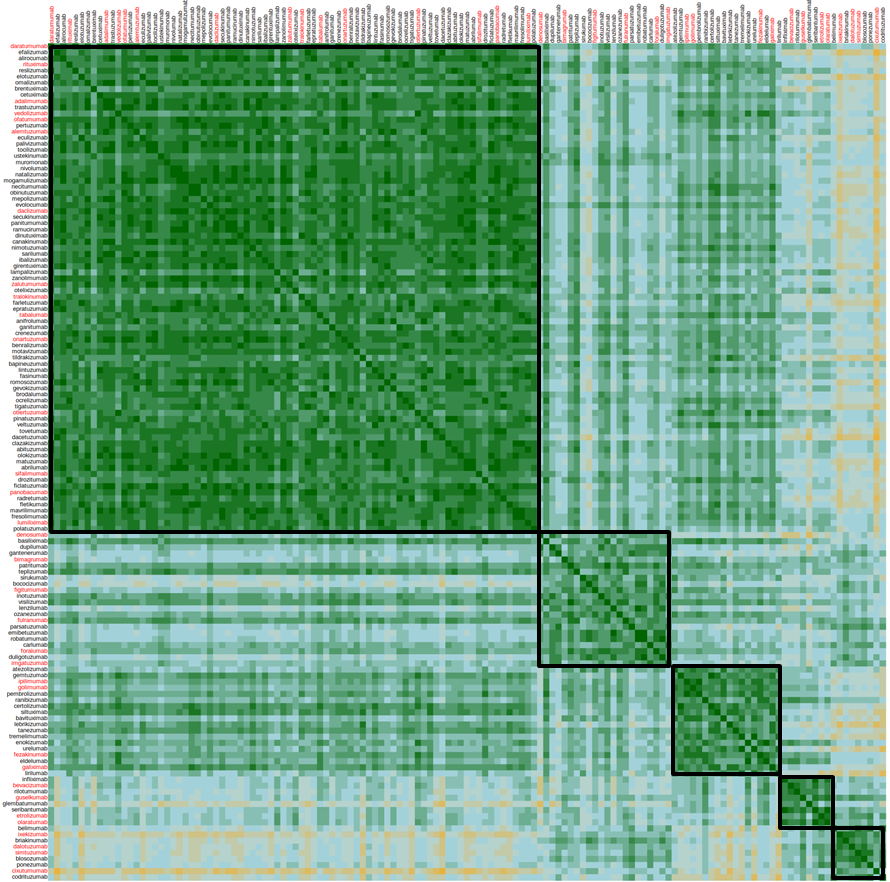
**

**Figure S1. Hierarchical clustering diagram showing groups of antibodies clustered by their biophysical properties in Jain et al..** Each black open box represents a cluster of mAbs, numbered 1–5 from top-left to bottom-right. Dark green squares indicate strong positive correlations, whilst yellow squares indicate poor correlation. The antibodies highlighted in red were selected to form the (Clinical)_33_ subset of proteins used in this study. More information on these mAbs can be found in Supplementary Tables I and II.

**Figure S2: Percentage of protein in pellet for the (Clinical)_33_ mAbs under quiescent conditions.** mAb solutions (0.5 mg mL^-1^ in 25 mM HEPES, 150 mM sodium chloride, pH 7.3) were incubated for 20 minutes at room temperature. Samples were then analyzed using the insoluble protein pelleting assay (Methods). Error bars show the range from two independent experiments. Antibodies are arranged (left to right) by increasing flow-induced aggregation, as in Figure 2a. Negative values (i.e. [soluble fraction] > [pelleted fraction]) arise due to experimental error under conditions where there is minimal aggregation. Technical and biological replicates show that the average error for this method is 3% for quiescent samples.

**
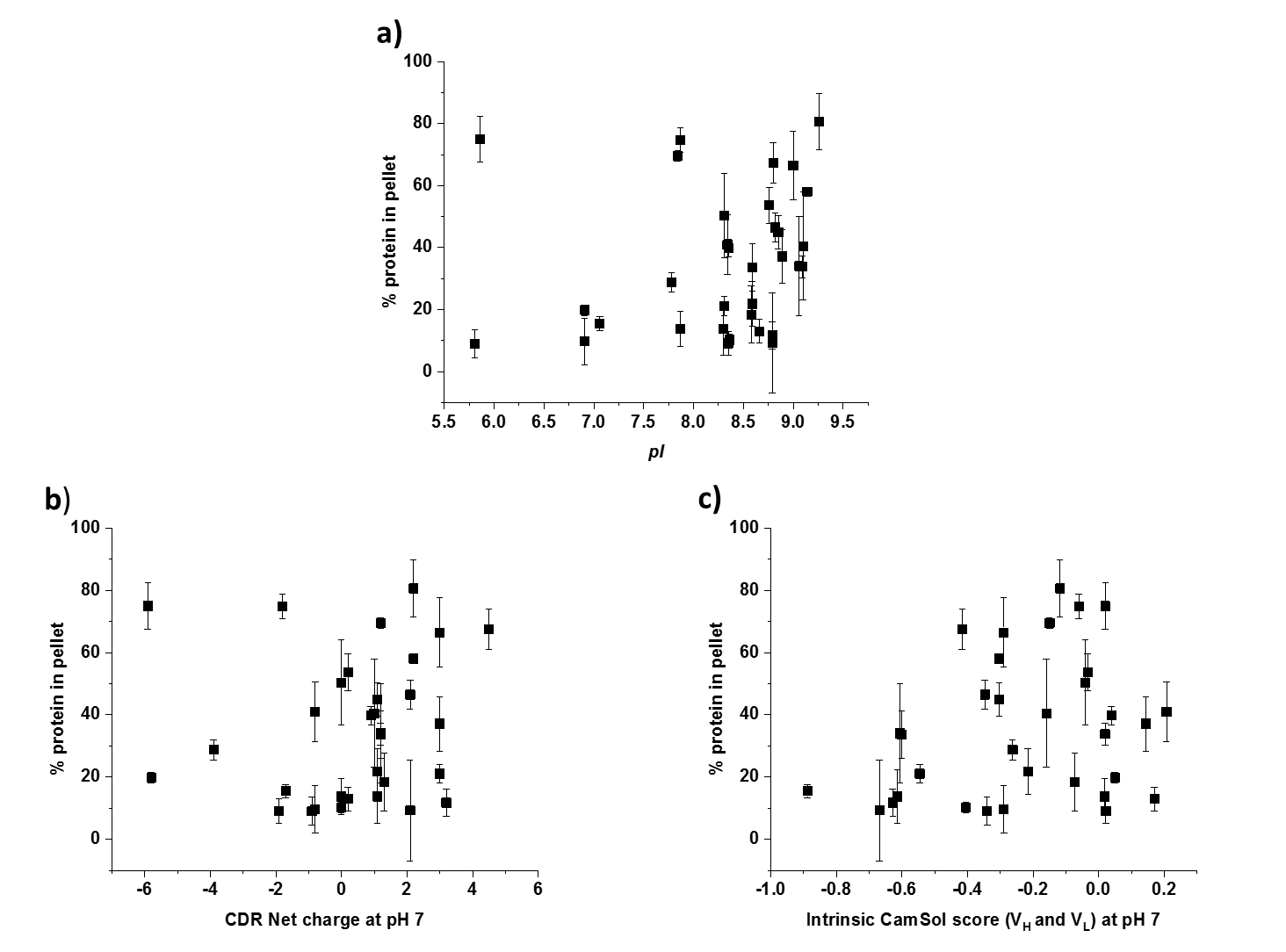
**

**Figure S3: Correlation of extensional flow-induced aggregation with: a) pI of the V_H_ and V_L_ domains, b) Net charge of CDRs at pH7 and c) Intrinsic CamSol score of V_H_ and V_L_ at pH 7 for the (Clinical)_33_ mAbs.** Error bars show the propagated error from two independent experiments. Spearman’s rank analysis was performed for each *in silico* metric against the flow data, yielding Spearman’s rank correlation coefficients of a) 0.32, b) 0.21 and c) 0.22 for Figures S3a, b and c, respectively.

**
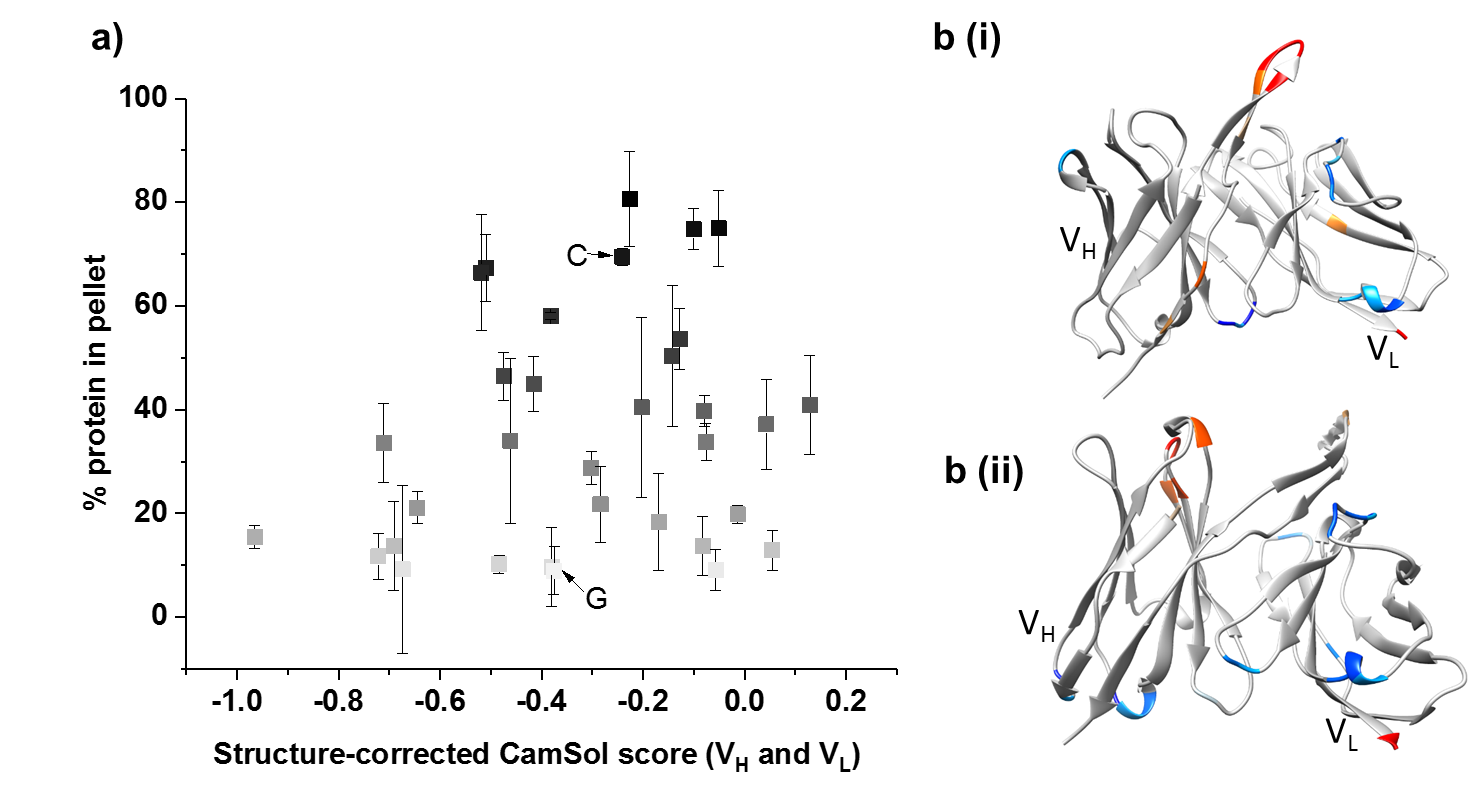
**

**Figure S4: Relationship between structure-corrected CamSol score and flow-induced aggregation data for the (Clinical)_33_.** a) Plot of the percentage of mAb pelleted by ultra-centrifugation after exposure to extensional flow versus structure-corrected CamSol scores computed for V_H_ and V_L_ domains at pH 7. Data points are shaded according to Figure 2a, with lighter shades denoting low levels of aggregation and darker shades corresponding to higher levels of aggregation. Regions predicted to drive insolubility from the native state would thus populate the bottom left quadrant of the plot. Error bars show the propagated error from two independent experiments. b) Representative homology models of (i) galiximab and (ii) cixutumumab. The V_H_ domain is shown on the left and the V_L_ domain on the right. Residues predicted by CamSol to be soluble (CamSol score ≥ +1) or insoluble (CamSol score ≤ -1), are highlighted in blue and red, respectively. The data points for these mAbs are denoted ‘G’ and ‘C’, respectively, in Figure S4a. Spearman’s rank analysis was performed on the data in Figure S4, yielding a Spearman’s rank correlation coefficient of 0.18.

**Figure S5: Stretch plot showing the relationship between TANGO and *ΔG^contrib^* values for the APRs of the (Clinical)_33_, in addition to the aggregation propensity of each mAb under flow** Data points are shaded according to Figure 2a, with lighter shades denoting low levels of aggregation and darker shades corresponding to higher levels of aggregation. Higher TANGO scores indicate an increased predicted aggregation propensity for that APR. *ΔG^contrib^* indicates the contribution of that APR to the thermodynamic stability of the protein, with values of *ΔG^contrib^* ≤ 0 being favorable. APRs that drive native-state aggregation would thus be predicted to populate the top right quadrant of the plot.


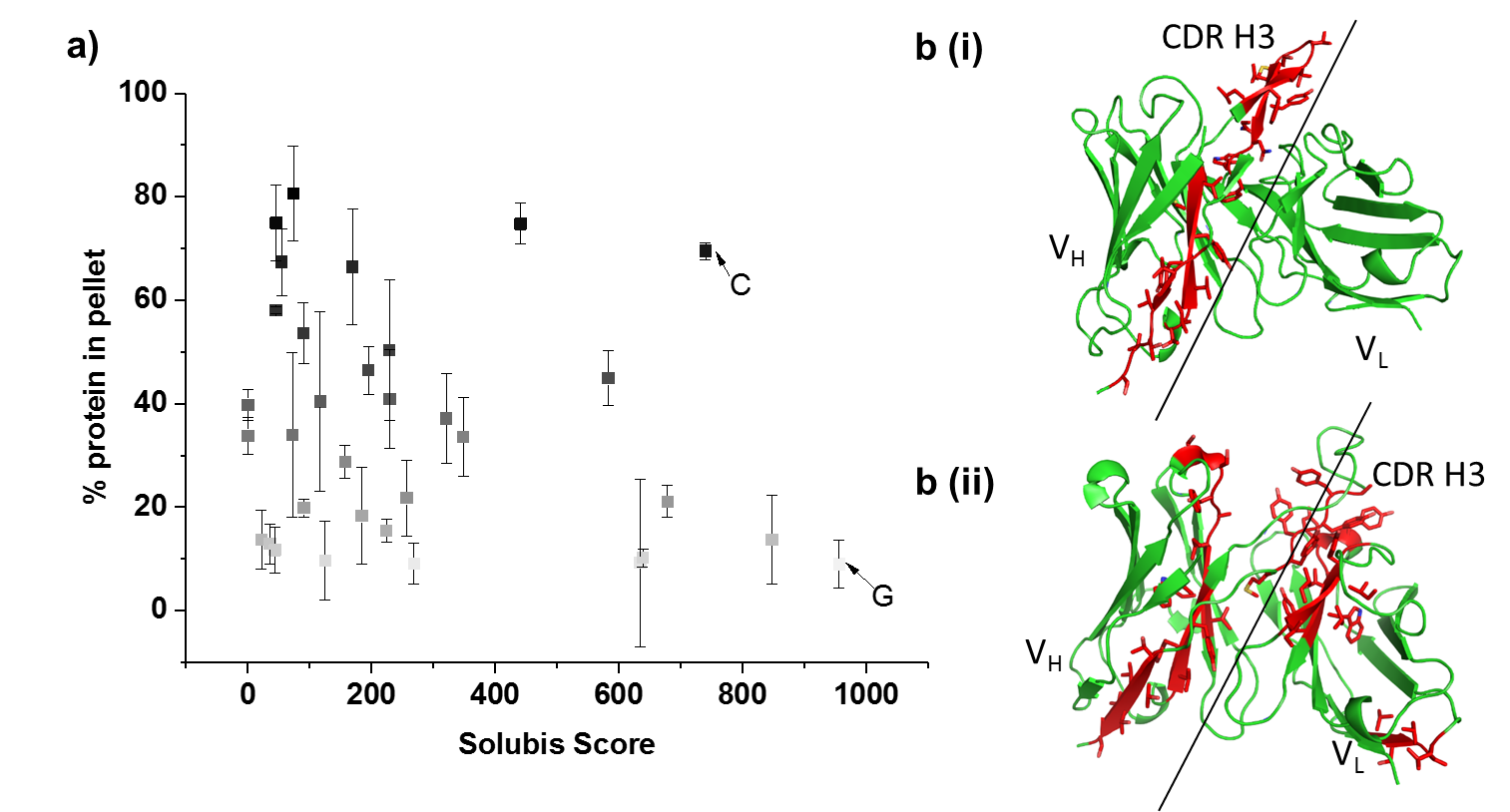


**Figure S6: Relationship between Solubis score and flow-induced aggregation for the (Clinical)_33_ mAbs.** a) Plot of the percentage of mAb that was pelleted by ultra-centrifugation after exposure to extensional flow against Solubis score for the 33 mAbs. Data points are shaded according to Figure 2a, with lighter shades denoting low levels of aggregation and darker shades corresponding to higher levels of aggregation. Error bars represent the propagated error from two independent experiments b) Homology models, generated using ABodyBuilder ^2^ of the V_H_ (left) and V_L_ (right) domains of (i) galiximab and (ii) cixutumumab. Aggregation-prone regions are highlighted in red, with the rest of the model colored in green. The V_H_-V_L_ interface is indicated by the black lines shown. The data points for these mAbs are denoted ‘G’ and ‘C’, respectively, in Figure S6a. Spearman’s rank correlation analysis was performed on the data in Figure S6, yielding a Spearman’s rank correlation coefficient of -0.19.


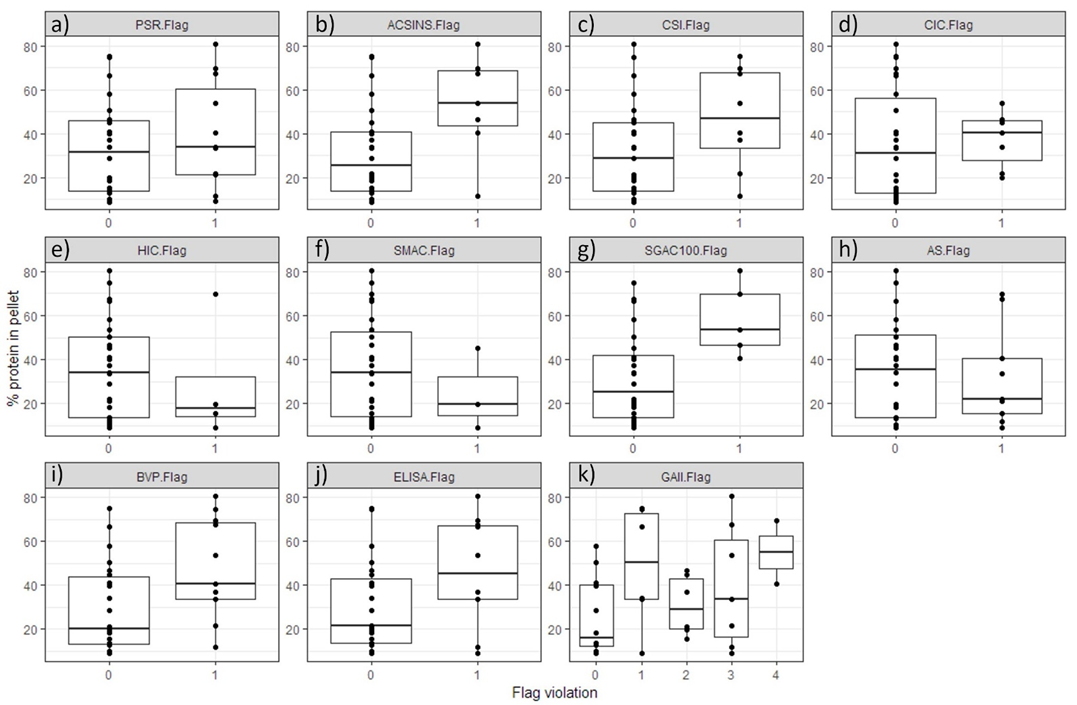


**Figure S7: Box plots comparing ten ‘developability’ assays from Jain et al.^1^ with the extensional-flow induced aggregation data obtained in this study.** For each assay conducted by Jain et al.^1^ the 33 mAbs used in this study were binned into two datasets: those which did not exceed the red flag threshold for that assay (0) and those that did (1). Box-plots show the wide distribution of flow-induced aggregation behavior for each dataset. The assays are: a) Polyspecificity reagent (PSR), b) Affinity capture, self-interaction nanoparticle spectroscopy (AC-SINS), c) Clone self-interaction (CSI), d) Cross-interaction spectroscopy (CIC), e) Hydrophobic interaction chromatography (HIC), f) Standup monolayer adsorption chromatography (SMAC), g) Salt-gradient AC-SINS (SGAC-SINS), h) monomer loss following accelerated stability (AS), i) Baculovirus particle (BVP) and j) Enzyme-linked immunosorbent assay (ELISA). k) box plot showing the total number of flags over assay groups^1^, acquired by a given mAb (GAll.Flag) against the percentage of mAb that was pelleted by ultra-centrifugation after exposure to extensional flow.

**Supplementary references**

(1) Jain, T.; Sun, T.; Durand, S.; Hall, A.; Houston, N. R.; Nett, J. H.; Sharkey, B.; Bobrowicz, B.; Caffry, I.; Yu, Y.; et al. Biophysical Properties of the Clinical-Stage Antibody Landscape. *Proc. Natl. Acad. Sci.* **2017**, *114* (5), 944–949. https://doi.org/10.1073/pnas.1616408114.

(2) Leem, J.; Dunbar, J.; Georges, G.; Shi, J.; Deane, C. M. ABodyBuilder: Automated Antibody Structure Prediction with Data–Driven Accuracy Estimation. *MAbs* **2016**, *8* (7), 1259–1268. https://doi.org/10.1080/19420862.2016.1205773.

(3) Gasteiger, E.; Hoogland, C.; Gattiker, A.; Duvaud, S.; Wilkins, M. R.; Appel, R. D.; Bairoch, A. Protein Identification and Analysis Tools on the ExPASy Server. In *The Proteomics Protocols Handbook*; 2005; pp 571–607. https://doi.org/10.1385/1592598900.

(4) Alam, M. E.; Geng, S. B.; Bender, C.; Ludwig, S. D.; Linden, L.; Hoet, R.; Tessier, P. M. Biophysical and Sequence-Based Methods for Identifying Monovalent and Bivalent Antibodies with High Colloidal Stability. *Mol. Pharm.* **2018**, *15* (1), 150–163. https://doi.org/10.1021/acs.molpharmaceut.7b00779.

(5) Sormanni, P.; Aprile, F. A.; Vendruscolo, M. The CamSol Method of Rational Design of Protein Mutants with Enhanced Solubility. *J. Mol. Biol.* **2015**, *427* (2), 478–490. https://doi.org/10.1016/j.jmb.2014.09.026.

(6) Pettersen, E. F.; Goddard, T. D.; Huang, C. C.; Couch, G. S.; Greenblatt, D. M.; Meng, E. C.; Ferrin, T. E. UCSF Chimera--a Visualization System for Exploratory Research and Analysis. *J. Comput. Chem.* **2004**, *25* (13), 1605–1612. https://doi.org/10.1002/jcc.20084.

(7) van der Kant, R.; Karow-Zwick, A. R.; Van Durme, J.; Blech, M.; Gallardo, R.; Seeliger, D.; Aßfalg, K.; Baatsen, P.; Compernolle, G.; Gils, A.; et al. Prediction and Reduction of the Aggregation of Monoclonal Antibodies. *J. Mol. Biol.* **2017**, *429* (8), 1244–1261. https://doi.org/10.1016/j.jmb.2017.03.014.

(8) Van Durme, J.; De Baets, G.; Van Der Kant, R.; Ramakers, M.; Ganesan, A.; Wilkinson, H.; Gallardo, R.; Rousseau, F.; Schymkowitz, J. Solubis: A Webserver to Reduce Protein Aggregation through Mutation. *Protein Eng. Des. Sel.* **2016**, *29* (8), 285–289. https://doi.org/10.1093/protein/gzw019.

(9) Fernandez-Escamilla, A.-M.; Rousseau, F.; Schymkowitz, J.; Serrano, L. Prediction of Sequence-Dependent and Mutational Effects on the Aggregation of Peptides and Proteins. *Nat. Biotechnol.* **2004**, *22* (10), 1302–1306. https://doi.org/10.1038/nbt1012.

(10) Schymkowitz, J.; Borg, J.; Stricher, F.; Nys, R.; Rousseau, F.; Serrano, L. The FoldX Web Server: An Online Force Field. *Nucleic Acids Res.* **2005**, *33* (Web Server), W382–W388. https://doi.org/10.1093/nar/gki387.

(11) Raybould, M. I. J.; Marks, C.; Krawczyk, K.; Taddese, B.; Nowak, J.; Lewis, A. P.; Bujotzek, A.; Shi, J.; Deane, C. M. Five Computational Developability Guidelines for Therapeutic Antibody Profiling. *Proc. Natl. Acad. Sci.* **2019**, *116* (10), 4025–4030. https://doi.org/10.1073/pnas.1810576116.
